# Ovarian Microcystic Stromal Tumor: A Case Report and Literature Review

**DOI:** 10.3389/fmed.2020.00058

**Published:** 2020-02-25

**Authors:** Lin Deng, Dingqing Feng, Jing Liang, Jie Luo, Bin Ling

**Affiliations:** ^1^China-Japan Friendship Hospital, Beijing, China; ^2^Graduate School of Peking Union Medical College, Chinese Academy of Medical Sciences, Beijing, China

**Keywords:** microcystic stromal tumor, ovary, immunophenotype, diagnosis, treatment

## Abstract

**Background:** Microcystic stromal tumor is a recently described subtype of ovarian tumor characterized by microcystic pattern and diffuse immunoreactivity for CD10, vimentin, and β-catenin and negative for EMA. However, its diagnostic criterion and standard treatment remain unclear.

**Case presentation:** We report a rare case of a left side microcystic stromal tumor with diameter about 7 cm in a 25-year-old female and summarize all cases of MCST reported in this study. The present patient underwent left ovarian tumor resection. Generally, the tumor was solid and cystic mixed. Immunohistochemically, the tumor was expressed CD10, WT1, cyclin D1 and vimentin, and nuclear immunoreactivity for β-catenin but negative for α-inhibin, calretinin, CK AE1/AE3, PLAP, SALL-4, CK7, P53, EMA, CD99, AFP, desmin, CgA, E-cadherin, and melanA.

**Conclusion:** Unilateral ovary, solid-cystic, and a larger than 4–8 cm pelvic mass without serious abdominal pain are its clinical features. The immunophenotype of vimentin+/CD10+/WT-1+/β-catenin+(nuclei)/cyclin D1+ is supportive of diagnosis. For these patients, unilateral oophorectomy dissection could be selected.

## Introduction

Ovarian microcystic stromal tumor (MCST) is a rare ovarian tumor that was first characterized by Irving and Young in 2009 (1). They reviewed 16 cases' discriminative histologic and immunohistochemical features and defined them as MCST, which was characterized by the following features: (a) a microcystic pattern and regions with lobulated cellular masses, sometimes with hyalinized fibrous stroma intervening; (b) an absence of morphologic features enabling any other specific diagnosis in the sex cord stromal category; (c) an absence of epithelial elements; (d) an absence of teratomatous or other germ cell elements; and (e) a distinctive immunophenotype of CD10+ /vimentin+/epithelial membrane antigen-. Daichi Maeda et al. subsequently reported 2 MCSTs with aberrant β-catenin nuclear accumulation and mutations of CTNNB1 (2). To date, rare cases of MCST have been reported all over the world. The clinical features and pathogenesis of MCST have not been firmly established. In the present study, we discussed the clinical, histopathological, and immunohistochemical aspects of an MCST case and compared them with the features described in the literature.

## Case Presentation

A 25-year-old woman, gravida 2 and para 2, visited her local hospital due to a pelvic mass discovered via health examination with no relevant past medical story, no abdominal discomfort, no complaints of menstrual disorder and no urination or defecation disturbance. She also denied a family history of cancer. Upon gynecological examination, a 7-cm mainly cystic component was discovered in the left adnexal area. B ultrasonography revealed a 7 cm^*^5.7 cm^*^3.9 cm increased left ovary with various cystic masses. The largest of them is 2.5 cm^*^1.8 cm. The serum levels of tumor markers, such as carcinoembryonic antigen (CEA), CA199, CA153, CA125, and a-fetoprotein (AFP), were within the normal range. The serum levels of total cholesterol were 5.50 mmol/L, triglyceride 2.21 mmol/L, apolipoprotein 1.01 g/L, specific beta hcG 0.10 mIU/ml, E2 144 pg/ml LH 7.74 mIU/ml, FSH 2.14 mIU/ml, PROG 11.54 ng/ml, PROL 75.4 ng/ml, and TESTO 0.48 ng/ml. Based on the chief complaint and imaging results, the diagnosis was considered as an ovarian chocolate cyst at first. Laparoscopic left ovarian tumor resection was subsequently performed at the local hospital. During laparoscopy, the surgeons discovered a 7 cm^*^6 cm cystic-solid mass and a 2-cm cystic mass present on the left increased ovary, and the uterus and right ovary were unremarkable. Frozen section of the left ovarian mass demonstrated an ovarian sex-stromal tumor, mostly like an ovarian granulosa cell tumor. After discussing treatment options with her family to balance curative treatment and fertility preservation and receiving her consent, the surgeons in the local hospital finally chose left ovarian tumor resection. No further oncologic therapy was administered.

Thirty days after surgery, the patient visited our hospital and Peking Union Medical College Hospital for pathology consultation and was diagnosed with ovarian microcystic stromal tumors. And till now, 4 months after surgery, the B ultrasonography and serum levels of tumor markers are all within the normal range.

## Pathologic Findings

### Materials and Methods

The resection specimens were processed on 4-μm slides and stained with haematoxylin and eosin in the local hospital. Immunochemical staining of inhibin-α, calretinin, cytokeratin AE1/AE3 (CK AE1/AE3), PLAP, SALL-4, CK7, WT1, P53, epithelial membrane antigen (EMA), CD99, AFP, vimentin, CD10, desmin, synaptophysin (Syn), CD56, chromogranin (CgA), Ki67, and LCA was performed in the local hospital. We reviewed the haematoxylin and eosin-stained and immunohistochemistry sections and performed β-catenin (Zsbio Store) and melanA (Zsbio Store) staining for diagnosis with the Ventana Benchmark XT automated staining system (Ventana Medical Systems, Inc., Tucson, AZ, USA) according to standard techniques. Peking Union Medical College Hospital subsequently added CD10, E-cadherin, cyclin D1 and Ki-67 staining, and reviewed all of the slides.

### Histological Findings

**Figure 1:**
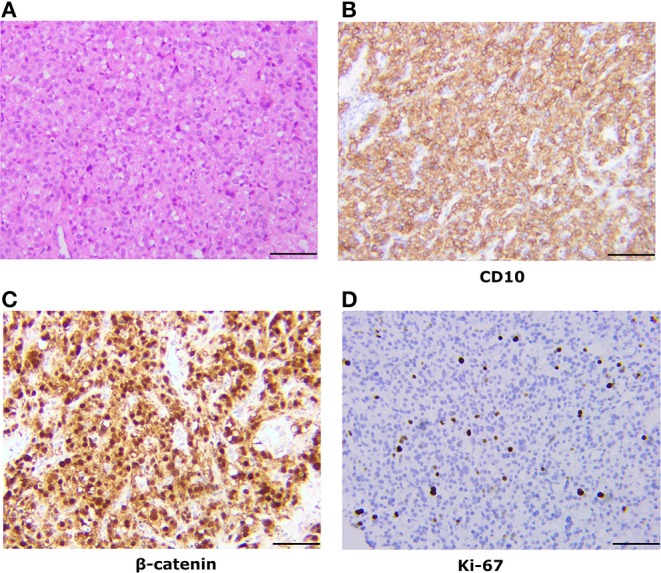
Pathologic findings of the ovarian tumor. **(A)** Histologically, the tumor was a solid-microcystic pattern and showed large, dark, round to ovoid nuclei. Nuclear atypia and mitotic figures were occasionally shown (H&E staining, x200). **(B)** Immunostaining reveals that the tumor cells are positive for CD10 (x200). **(C)** Immunostaining reveals that the tumor cells are nuclear positive for β-catenin (x200). **(D)** Immunostaining shows a low Ki-67 proliferation index.

The tumor was confined to the left ovary and had a smooth outer surface. It measured 7 cm in maximum diameter and revealed a solid-cystic mixed appearance. Microscopically, the tumor consists of diffuse cells, with large, darkly variable sizes, round, oval, and fusiform nuclei (Figure 1A). Tumor giant cells and bizarre nuclei could be noted in this tumor, and mitotic figures were occasionally shown.

Another small cyst was also fixed in the left ovary, measuring 2 cm at the greatest dimensions. The cyst wall consisted of corpus luteum tissues with blood and infrequent tumor tissues.

### Immunohistochemistry

The tumor cells commonly showed diffuse and strong cytoplasmic membranous expression for CD10, WT1, cyclin D1, and vimentin and nuclear immunoreactivity for β-catenin (Figures 1B,C). Cells were negative for α-inhibin, calretinin, CK AE1/AE3, PLAP, SALL-4, CK7, P53, EMA, CD99, AFP, desmin, CgA, E-cadherin, and melanA. Tumor cells were focally positive for Syn and CD56. The Ki-67 proliferation index was low (6%) (Figure 1D).

### Pathologic Diagnosis

This patient was initially diagnosed with a sex cord stroma tumor at her local hospital. Subsequently, the histopathological diagnosis was revised and confirmed as MCST of the ovary by the authors and two experienced pathologists (Prof. Luo in China Japan Friendship Hospital and Prof. Guo in Peking Union Medical College Hospital).

## Discussion

This case is a very rare tumor of ovarian MCST, which is a newly categorized ovarian tumor first described by Irving and Young in 2009 (1). To date, rare cases have been reported all over the world. This neoplasm exhibits some distinctive morphological and immunochemical features. In most cases, the tumor has variable islands of monotonous round to ovoid cells with microcystic spaces and has diffusely strong immunoreactivity for CD10, vimentin, WT-1, and β-catenin (nuclei) and is negative for EMA (1–13). These features are readily apparent and permit the correct diagnosis. Consistent with previous reports, the tumor in our study was diffusely strongly positive for CD10, WT1, vimentin, and β-catenin (nuclei) and negative for α-inhibin, calretinin, EMA, and E-cadherin. We also found it diffusely strongly positive for cyclin D1, partially positive for Syn and CD56, and negative for CK(AE1/AE3), PLAP, SALL-4, CK7, P53, EMA, CD99, AFP, desmin, CgA, and melanA.

**Figure 2:**
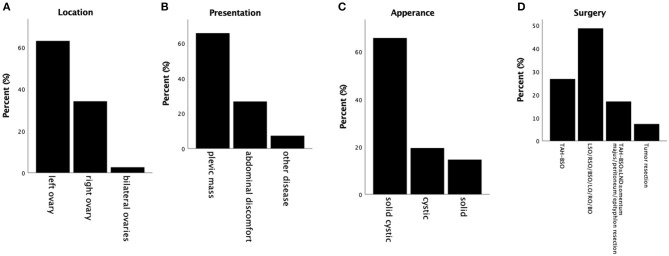
Summary of clinical profiles of MCSTs reported. **(A)** Tumor location. The majority of tumors are unilateral, and most are fixed on the left side. **(B)** Presentations reported of these cases. Pelvic mass is taken as the most commonly presented symptom. **(C)** Appearances of these tumors. Most of these tumors are solid-cystic. **(D)** The surgery status of MCSTs. Almost all of these patients underwent oophorectomy, and more than half of them underwent salpingo dissection with or without additional hysterectomy at the same time.

However, given the rarity of this tumor and the limited investigation of the clinical and immunohistochemical profile, we compared our results with others reported and summarized their similarities. The clinical features of 45 cases of ovarian MCST have been summarized (Table 1) (1–13). As some cases in the two studies reported by Irving in 2009 and 2011 were repeated and lacked details, we only recorded the mean values they afforded in the literature. According to our analysis, MCST occurs in adult women with a mean age of 44 years (range 24–71). Almost all of the tumors were unilateral except 1 bilateral, and the left side (24/42 cases) was more frequently involved than the right side (13/42 cases) (8) (Figure 2A). Three cases were not mentioned. The patients most commonly presented with a pelvic mass (27/42 cases, 64.3%) and abdominal discomfort (11/42 cases, 26.2%) (Figure 2B). Only 3 patients presented with other diseases, 1 case of cervical disease (10), 1 case of dysfunctional uterine bleeding (1), 1 case of endometrial disease (8) and 1 case without presentation. Generally, the tumors were mixed solid and cystic (27/42 cases, 64.3%), solid (6/42 cases, 14.3%), and cystic (8/42 cases, 19%) (Figure 2C). The remaining 1 case was not mentioned in the report. The sizes of the tumors ranged from 1 to 27 cm (mean 9.86 cm). Half of these tumors were larger than 8 cm, and the majority (93%) were larger than 4 cm. Limit cases had detected the tumor serum levels. The CA125 level was evaluated in 17 cases, of whom 4 cases had elevated levels (6, 11–13). In this case we reported, tumor serum levels before surgery were all within the normal range. And 4 months after surgery, the serum levels were still within the limit. The CA199 level of all cases detected was within the limit. CA125 and CA199 might not have significance for MCST patients' prognosis prediction, especially in patients whose serum levels are within normal range before surgery. Most of the patients elect to at least dissect their affected ovary. A total of 47.6% of patients underwent oophorectomy with or without salpingectomy, and 42.9% of patients underwent total abdominal hysterectomy and bilateral salpingo oophorectomy with or without lymph node dissection, omentectomy, and appendectomy (Figure 2D). Two of the patients initially received gonadotropin-releasing hormone analogs before surgery (2, 10). However, both of these patients presented with increased tumor size after treatment. Data for clinical follow-up were obtained for 20 patients and ranged from 1 to 150 months from the time of initial diagnosis. Therefore, we regard unilateral ovary, solid-cystic, and a larger than 4–8 cm pelvic mass without serious abdominal pain or abnormal serum levels of tumor makers as the clinical features of MCST. According to the follow-up profile of cases reported, additional hysterectomy will not improve the prognosis compared with unilateral oophorectomy with or without salpingo dissection. And if serum levels of tumor markers are all within the normal range before surgery, further re-inspection after the surgery has no significance for prognosis prediction.

**Table 1:** Clinical features.

**References**	**Case**	**Tumor location**	**Clinical presentation**	**Surgery status**	**Imaging finding**	**Age (year)**	**Tumor size (cm)**	**Follow up time (m)**
Irving and Young (1)	1	Left ovary	Plevic mass	TAH-BSO, LND, omentum majus	Solid-cystic	62	27	NK
	2	Left ovary	Abdominal discomfort	TAH-BSO, peritoneum sampling	Solid-cystic	45	10	NK
	3	Left ovary	Plevic mass	TAH-BSO, omentum majus, epityphlon	Solid-cystic	51	12	NK
	4	Left ovary	Plevic mass	LO	Multilocular cystic	29	10	NK
	5	Right ovary	Plevic mass	TAH-BSO, LND, peritoneum sampling	Unilocular cystic	58	6.2	NK
	6	NS	Abdominal pain	BSO	Solid-cystic	26	8.5	NK
	7	Right ovary	Plevic mass	RO	Solid-cystic	29	6	NK
	8	Left ovary	Plevic mass	TAH-LSO	Solid	45	4	NK
	9	Right ovary	Plevic mass	RO	Solid-cystic	63	4.6	NK
	10	NS	Plevic mass	BSO	Solid-cystic	56	4.2	NK
	11	Right ovary	Plevic mass	TAH-BSO	Solid-cystic	45	4.5	NK
	12	Left ovary	Plevic mass	TAH-BSO	Solid-cystic	55	24	NK
	13	Left ovary	Plevic mass	TAH-BSO	Solid-cystic	44	7	NK
	14	Left ovary	Plevic mass	LSO	Solid-cystic	36	3	NK
	15	Right ovary	DUB	TAH-BSO	Solid	37	2	NK
	16	Right ovary	Plevic mass	LSO	solid	39	6.4	NK
Daichi Maeda et al. (2)	17	Right ovary	Plevic mass	1GnRHa—RSO, omentum majus	Solid-cystic	33	11.5	14 m
	18	Right ovary	Abdominal discomfort	BSO	Multilocular cystic	41	9.5	4 m
Yang et al. (13)	19	Left ovary	Abdominal pain	Tumor resection	Solid-cystic	45	16	NK
Irving et al. (4)	20–23(11 cases reported in 2009 + 4 new cases)	NK	NK	NK	NK	29-63, mean 43	mean7.3	NK
Kang et al. (5)	24	Left ovary	Abdominal discomfort	LSO	Solid	41	7.8	NK
Lee et al. (7)	25	Left ovary	Pelvic mass	LSO, right ovary partial resection, colon resection	Solid-cystic	40	15	NK
Bi et al. (3)	26	Left ovary	Pelvic mass	LSO	Solid-cystic	69	15	60 m
	27	Left ovary	Pelvic mass	LSO + right ovary sampling	Solid-cystic	29	5.5	18 m
	28	Left ovary	Pelvic mass	LO	Solid-cystic	40	8	7 m
	29	Left ovary	Pelvic mass	TAH-BSO	Multilocular cystic	65	11	NK
	30	Left ovary	Pelvic mass	TAH-BSO	UNILOCULAR cystic	57	10	59 m
	31	Left ovary	Pelvic mass	TAH-BSO, omentum majus, epityphlon	Unilocular cystic	41	7	2 m
Podduturi et al. (12)	32	Right ovary	Abdominal discomfort	TAH-BSO, LND, omentum majus	Solid-cystic	50	14	NK
Lee et al. (6)	33	Left ovary	Abdominal discomfort	LSO	Multilocular cystic	24	18	NK
	34	Left ovary	Pelvic mass	LSO, LND, peritoneum	Solid-cystic	31	24	NK
Murakami et al. (10)	35	Left ovary	Cervical disease	4GnRHa—LSO	Solid-cystic	26	6	36 m
Na et al. (11)	36	Right ovary	Pelvic mass	RSO + omentum majus	Solid-cystic	33	8.6	57 m
	37	Left ovary	Abdominal pain	LSO + LND sampling	Solid-cystic	31	24	20 m
McCluggage et al. (8)	38	NS	Pelvic mass	BSO	Solid-cystic	61	NS	NK
	39	Right ovary	Thicken endometrium	TAH-BSO	solid-cystic	56	1	NK
	40	Bilateral ovaries	Pelvic mass	TAH-BSO	Solid	45	7	NK
	41	Right ovary	Pelvic mass	BSO	Solid	71	4	NK
Meurgey et al. (9)	42	2 left ovary, 1 right ovary	Abdominal discomfort	2LSO, 1RSO	Solid-cystic	46	7.5–11, mean 9.25	NK
	43		Abdominal discomfort		Solid-cystic	37		NK
	44		Abdominal discomfort		Unilocular cystic	47		NK
Lin Deng et al. (this case)	45	Left ovary	Pelvic mass	Tumor resection	Solid-cystic	25	7	1 m

*DUB, Dysfunctional uterine bleeding; BSO, Bilateral salpingo oophorectomy; LND, Lymph node dissection; LO, Left oophorectomy; LSO, Left salpingo oophorectomy; RO, Right oophorectomy; RSO, Right salpingo oophorectomy; NK, Not known*.

**Figure 3:**
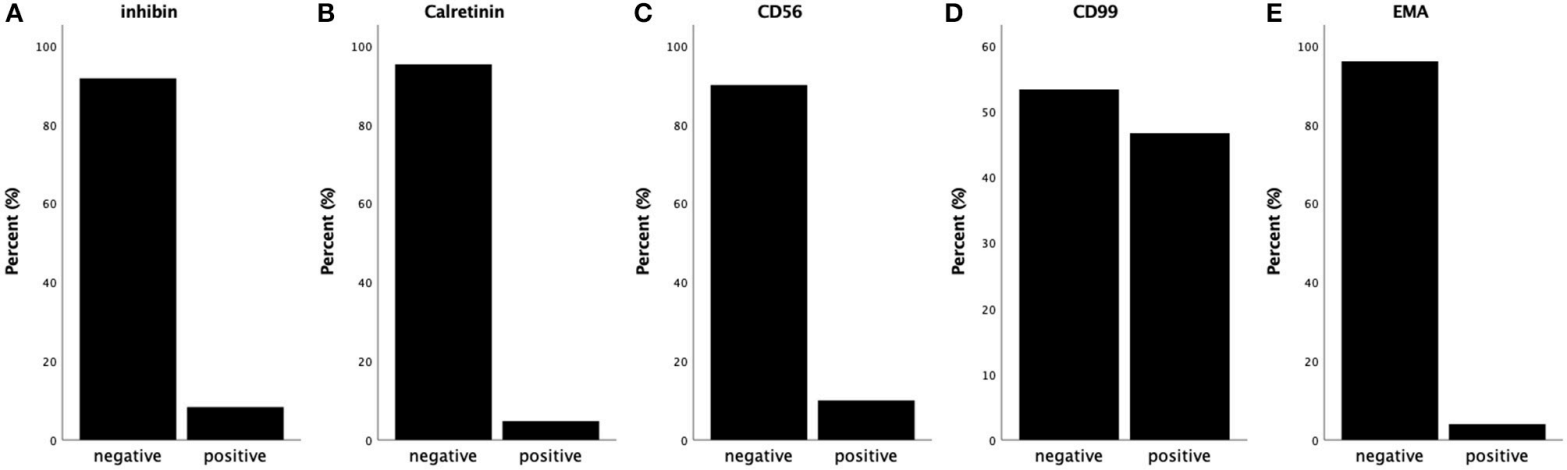
Summary of immunochemical features. **(A)** Almost all of the tumors are negative for α-inhibin. **(B)** Almost all of the tumors are negative for calretinin. **(C)** The majority of the tumors are negative for CD56 (90%). **(D)** Almost half of the tumors are negative for CD99, and approximately 47% are positive. **(E)** Almost all of the tumors are negative for EMA.

Immunochemical features of all 50 cases are summarized (Figure 3) (1–13). All tumors detected were strongly diffusely positive for vimentin, β-catenin, CD10, WT-1 and cyclin D1, and negative for melanA, PR, ER, PLAP, SALL4, desmin, CgA, E-cadherin, CK (AE1/AE3), Syn and CK7 in virtually 100% of tumor cells. Almost all tumors detected were negative for α-inhibin (22/24 cases, 91.7%) and calretinin (20/21 cases, 95.2%) (Figures 3A,B). CD 56 was negative in 9 cases and positive in 1 case (Figure 3C). CD99 was positive in 7 cases and negative in 8 cases (Figure 3D). EMA was negative in 24 cases and positive in 1 case (Figure 3E). The remaining cases were not mentioned within these reports. Among these markers, CD10, α-inhibin and calretinin are markers for sex cord-stromal tumors; CK (AE1/AE3), while EMA and E-cadherin are markers for epithelial tumors. Tumors that most often enter the differential diagnosis with MCSTs are tumors that, like thecomas, steroid cell tumors and sclerosing stromal tumors, have some similarities with MCST. MCST, which is diffusely strongly positive for CD10 and lacks α-inhibin and calretinin, is distinct from the neoplasms mentioned, which are positive for α-inhibin and calretinin. Cyclin D1 is a β-catenin-regulated oncogene that is often co-expressed with β-catenin in many tumors. Recent studies have indicated that the Wnt/β-catenin signaling pathway is the key pathway in tumourigenesis. Co-expression of cyclin D1 and β-catenin in the present study provides further evidence to support this hypothesis. CD10, vimentin and WT-1 positivity have been reported in various kinds of sex cord stromal tumors as well, but an ovarian tumor that expresses nuclear β-catenin and the combination of CD10, vimentin and cyclin D1 is unique. Therefore, we regard this vimentin+/CD10+/WT-1+/β-catenin+(nuclei)/cyclinD1+/EMA-/α-inhibin-/calretinin-/ E-cadherin- immunophenotype as an immunochemical key feature that characterized ovarian MCST. The β-catenin (CTNNB1) mutation causes deregulation of β-catenin degradation and results in β-catenin nuclear accumulation.

## Conclusions

We presented a rare case of MCST and summarized the distinct clinical, histological, and immunohistochemical features of all the cases reported. Pathologists and clinicians should be aware of the existence of this unique neoplasm, unilateral ovary, solid-cystic, larger than 4–8 cm pelvic mass without serious abdominal pain. Serum levels of tumor markers are always within the limits; sometimes, CA125 may be elevated. The immunophenotype of vimentin+/CD10+/WT-1+/β-catenin+(nuclei)/cyclinD1+/EMA-/α-inhibin-/calretinin-/ E-cadherin- is supportive of diagnosis as MCST. For these patients, unilateral oophorectomy with or without salpingo dissection is a good choice. The findings presented here provide insights into this tumor and will facilitate future studies. In addition, further research is needed regarding the hormone levels, prognosis and tumourigenic mechanisms of these tumors.

## Data Availability Statement

The datasets used and analyzed during the current study are available from the corresponding author on reasonable request.

## Ethics Statement

Written informed consent was obtained from the participant for the publication of this case report. Ethical approval was given by the Medical Ethics Committee of the China Japan Friendship Hospital.

## Author Contributions

LD, BL, and JL diagnosed the patient. LD and JL carried out experiments. LD analyzed the experimental results. All authors wrote and revised the manuscript.

### Conflict of Interest

The authors declare that the research was conducted in the absence of any commercial or financial relationships that could be construed as a potential conflict of interest.

## Abbreviations

MCST: Microcystic Stromal Tumor
CEA: carcinoembryonic antigen
AFP: a-fetoprotein
DUB: Dysfunctional uterine bleeding
BSO: Bilateral salpingo oophorectomy
LND: Lymph node dissection
LO: Left oophorectomy
LSO: Left salpingo oophorectomy
RO: Right oophorectomy
RSO: Right salpingo oophorectomy
NK: Not known.
